# Genetic relationships and identification of core germplasm among rice photoperiod- and thermo-sensitive genic male sterile lines

**DOI:** 10.1186/s12870-021-03062-x

**Published:** 2021-07-02

**Authors:** Xianwen Zhang, Qiang He, Wuhan Zhang, Fu Shu, Weiping Wang, Zhizhou He, Hairong Xiong, Junhua Peng, Huafeng Deng

**Affiliations:** 1 College of Bioscience and Biotechnology, Hunan Agricultural University, Changsha, 410128 China; 2 State Key Laboratory of Hybrid Rice, Hunan Hybrid Rice Research Center, Hunan Academy of Agricultural Sciences, Changsha, 410125 China; 3 Huazhi Biotech Co. Ltd, Changsha, 410125 China; 4 School of Chemistry and Materials Science, Hunan Agricultural University, Changsha, 410128 China

**Keywords:** Male sterility, Two-line hybrid, Genetic diversity, PTGMS

## Abstract

**Background:**

Harnessing heterosis is one of the major approaches to increase rice yield and has made a great contribution to food security. The identification and selection of outstanding parental genotypes especially among male sterile lines is a key step for exploiting heterosis. Two-line hybrid system is based on the discovery and application of photoperiod- and thermo-sensitive genic sensitive male sterile (PTGMS) materials. The development of wide-range of male sterile lines from a common gene pool leads to a narrower genetic diversity, which is vulnerable to biotic and abiotic stress. Hence, it is valuable to ascertain the genetic background of PTGMS lines and to understand their relationships in order to select and design a future breeding strategy.

**Results:**

A collection of 118 male sterile rice lines and 13 conventional breeding lines from the major rice growing regions of China was evaluated and screened against the photosensitive (*pms3*) and temperature sensitive male sterility (*tms5*) genes. The total gene pool was divided into four major populations as P1 possessing the *pms3*, P2 possessing *tms5,* P3 possessing both *pms3* and *tms5* genes, and P4 containing conventional breeding lines without any male sterility allele. The high genetic purity was revealed by homozygous alleles in all populations. The population admixture, principle components and the phylogenetic analysis revealed the close relations of P2 and P3 with P4. The population differentiation analysis showed that P1 has the highest differentiation coefficient. The lines from P1 were observed as the ancestors of other three populations in a phylogenetic tree, while the lines in P2 and P3 showed a close genetic relation with conventional lines. A core collection of top 10% lines with maximum within and among populations genetic diversity was constructed for future research and breeding efforts.

**Conclusion:**

The low genetic diversity and close genetic relationship among PTGMS lines in P2, P3 and P4 populations suggest a selection sweep and they might result from a backcrossing with common ancestors including the pure lines of P1. The core collection from PTGMS panel updated with new diverse germplasm will serve best for further two-line hybrid breeding.

**Supplementary Information:**

The online version contains supplementary material available at 10.1186/s12870-021-03062-x.

## Background

Rice (*Oryza sativa* L.) is the most important cereal and staple food for more than 50% of global population and 90% of the world’s rice is grown in Asia [1]. The world population is expected to rise to 10 billion by 2050 and to feed this population agriculture production needs to be expanded by 70%. It was estimated that the world population would require 763 million tons of rice in 2020, and 852 million tons by 2035 [2, 3]. Harnessing heterosis is one of the major approaches to increase rice yield and has made a great contribution to food security in China and many other countries [2, 4]. The hybrid rice production systems as three-line hybrid rice (cytoplasmic male sterile, CMS) and two-line hybrid rice (photoperiod- and thermo-sensitive genic male sterility) are based on the male sterility. AnnongS-1 was the first *indica* temperature sensitive genic male sterile line (TGMS) developed in China, which opened a new way of heterosis utilization in rice [5]. The two line hybrid system is based on the discovery and application of environmentally sensitive genic male sterile (EGMS) materials. Compared with three-line hybrid system, it has obvious technical advantages of easy fertility restoration and requires lesser time and labor in breeding hybrid rice lines [4, 6, 7]. The photo or temperature sensitive genetic male sterile (PTGMS) lines have occupied millions of hectares of rice field in China for more than a decade [4]. Despite the wide range usage of PTGMS lines in China, the knowledge of their genetic components is lacking [4]. Hence, it is valuable to ascertain the genetic background of PTGMS lines and to understand their relationships in order to select and design a future breeding strategy.

The identification and selection of outstanding parental genotypes especially among male sterile lines is a key step for exploiting heterosis. As per previous studies, the majority of cultivated rice varieties are resulted from hybrid rice combinations with wild abortive cytoplasm, which accounted for 69 and 6.8% of total rice cultivated area in China and India, respectively [6, 8, 9]. The monophyletic cytoplasm is likely to result in the genetic vulnerability. Therefore, the identification and utilization of excellent male sterile lines with new cytoplasm and broader genetic diversity should be the major task to achieve. Marker assisted selection (MAS) could be the best tool to select the plants based on their genotype.

Next generation sequencing (NGS) technology is now available for both plant biology research and plant breeding [10]. Various studies have been conducted to determine the genetic diversity, relationship, population structure, heterotic grouping, genes and quantitative trait locus (QTL) mapping in rice germplasm, including inbred lines using different genotyping platforms and marker density [11–15]. Recently, various traits of isonuclear-alloplasmic male sterile rice lines, including male sterility, agronomic characters, cytoplasmic effects, and resistance to different stresses have also been studied [8]. However, few reports were based on the differentiation of their nuclear genomes.

Currently, single nucleotide polymorphism (SNP)-chip technique is one of the fastest and accurate approaches of plant genotyping as compared to other molecular markers. SNP markers are highly abundant, amendable to high-throughput genotyping, and useful for a number of breeding and genetic applications in crops, including rice [16]. It enables researchers to readily select sub-sets of informative SNPs for use on smaller, in-house platforms for immediate applications in various genomic and molecular approaches including marker-assisted or genomic selection, genome-wide polymorphisms in high throughput QTL and association mapping. It is also used to select highly targeted sets of SNPs for high-resolution haplotype analysis and gene discovery. Several medium-density and high-density chip arrays have been developed for rice [17]. Theses SNP assays have been developed at different densities, for example the 50 K-SNP chip [18], C6AIR [19], the RICE6K [20], the 44 K-SNP chip [21], and the 700 K-SNP High Density Rice Array [22]. The SNP density required to meet these criteria in rice is ~ 6–7000 markers, due to the significant differences in SNP distribution and frequency that characterize the deeply differentiated subpopulations of *O. sativa* [19]. The availability of high-density SNP chips for rice makes it possible to undertake large-scale, high-throughput germplasm characterization, enhancing the value of the genetic resources available in the world’s major germplasm repositories.

In this study, 131 two-line PTGMS rice lines were clustered on the basis of sterile genes. The clusters were further genotyped by 56 K whole genome rice SNP-chip and the genetic relationship among the materials was analyzed. The core breeding collection of these materials was screened and the selective sweep that occurred during the breeding process was investigated. The results provide important insights into the narrow genetic basis of available PTGMS gene pool and is a reference for the future two-line hybrid breeding and research programs in rice.

## Results

### Screening for photo- and temperature-sensitive nuclear genes

The collection of 131 two-line male sterile lines was evaluated and screened against the photosensitive male sterility (*pms3*) and temperature sensitive male sterility (*tms5*) genes to identify the similarity among them. The total gene pool was divided into four major populations. There were 9.16% (12) lines in the population 1 (P1), possessing the *pms3* and denoted as the photosensitive genic male sterile population. A total of 77% (101) lines were in the population 2 (P2) possessing *tms5* and denoted as the thermo sensitive genic male sterile population*.* Only 3.82% (5) lines possessed both *pms3* and *tms5* genes and were classified as the population 3 (P3). They are denoted as the photo/thermo sensitive genic male sterile population. The other 9.92% (13) lines were the normal breeding lines without any PTGMS gene, classified in the population 4 (P4) and denoted as the conventional breeding population (Additional Table 1).

### Genotyping and evaluation of genomic variants

**Fig. 1:**
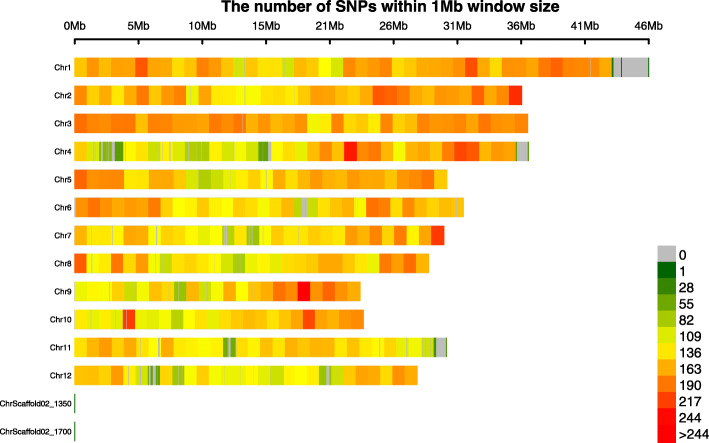
SNP markers density per Mb in the 12 chromosomes of rice genome genotyped by using 56 K SNP-Chip for 118 PTGMS lines and 13 conventional breeding lines

In the present study, we genotyped the 131 lines with 56 K SNP marker chip. The average SNP density was 1.52 SNPs per 10 kb genomic region with a range from 1.32 in the chromosome 12 to 1.74 SNP in the chromosome 3. The average filtered SNP density was one SNP per 15.5 kb genomic region which varied from 1 SNP per 9.7 kb for P4 to 26 kb for P3 (Fig. 1). The genome coverage ranged from 91% for chromosome 1 to 100% for most of the other chromosomes. The markers density was much higher than 12 SNPs per Mb in intergenic SNP based assay [19] but relatively lower than the previously reported 0.745 per kb 50 kb density in gene single copy-based chip [23]. Hence, the markers density in this study is suitable to investigate the genetic diversity among genotypes.

### Genetic purity

**Fig. 2:**
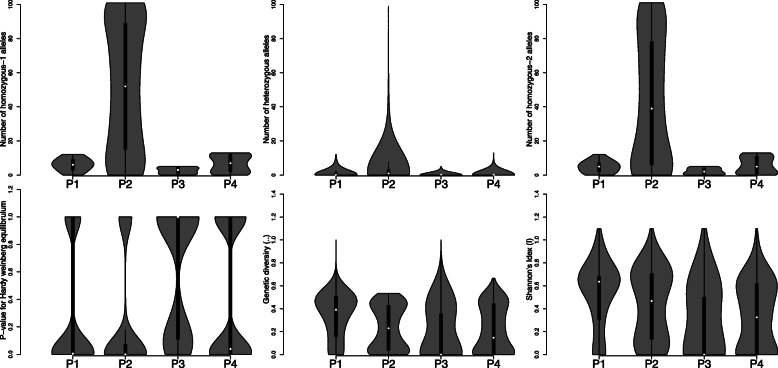
The trend and relationship among four populations for various genetic diversity parameters, including Number homozygous-1 alleles, Number of heterozygous alleles, Number homozygous-2 alleles, *P*-values for the Hardy Weinberg equilibrium, Genetic diversity parameter (π), and Shannon’s diversity index (I). The populations (P1-P4) were defined based on the presence of photosensitive male sterility (*pms3*) and temperature sensitive male sterility (*tms5*). P1 has *pms3*, P2 has *tms5*, P3 has both *pms3* and *tms5*, and P4 is the conventional breeding lines without male sterility genes

Genetic purity among the genotypes can be evaluated by the available homozygous or heterozygous alleles. In the current study, we assessed the frequency of homozygous and heterozygous alleles per locus and estimated the nucleotide diversity and Shannon’s index (I). The genome of all four populations was highly homozygous with 93 to 97% homozygous alleles. The lowest population heterozygosity among alleles was observed in P4 (2.66%) followed by P3 (3.14%), while the maximum heterozygosity was observed for P1 (6.80%) followed by P2 (3.70%) (Fig. 2, Additional Table 1).

### Genome wide nucleotide diversity

The genetic diversity among 118 male sterile lines and 13 conventional breeding lines was evaluated. The population’s nucleotide diversity (π) was low, ranging from 7.54 × 10^− 8^ to 4.29 × 10^− 4^. It indicates the close relatedness among the genotypes and suggests the limited number of available signature-genes in the germplasm. Among all four populations, we observed the lowest genome-wide *π*-value in the P4 (male fertile) genotypes (*π* = 0.0000339), while the highest genetic diversity (*π* = 0.0000503) was in P1. The values for genetic diversity among P2 (*π* = 0.0000348) and P3 (*π* = 0.0000347) were approximately same (Fig. 2, Additional Table 1). Within each population, the widest genetic diversity range was observed for P3, while it was the lowest in P2.

### Genome-wide genetic differentiation

**Fig. 3:**
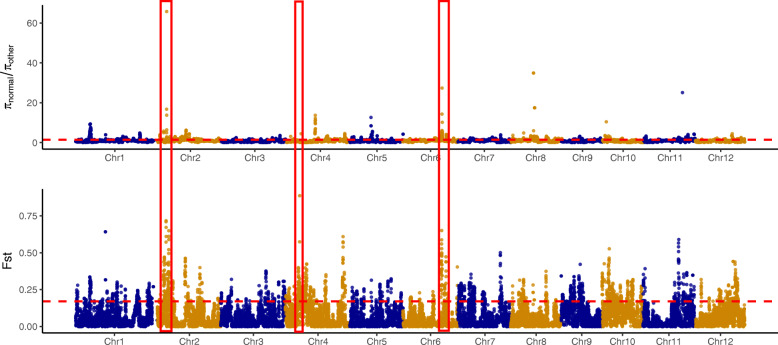
The genetic differentiation evaluated by FST values and the ratio of π-values among P4 versus others male sterile populations (P1, P2, P3). The red boxes in Chromosomes (Chr) 2, 4, and 6 indicate the top selective sweeps

Although the genetic differentiation between the whole male sterile population (P1, P2, P3) and the conventional lines was not very high (weighted Fst = 0.078), the highest levels (mean weighted Fst > 0.2) of genetic differentiation was revealed by P1 with other populations. It was maximum with P4 (weighted Fst = 0.252) followed by P3 (weighted Fst = 0.178) and P2 (weighted Fst = 0.108) (Fig. 3, Table 1). The level of genetic differentiation between P3 and P4 was similar to that of P1 from P2. On the other hand, the genetic differentiation of P2 from P3 and P4 was not very high (weighted Fst = 0.045 and 0.099, respectively). It suggests a driving force of *pms3* and *tms5* genes in shaping the genetic variation pattern and indicates the genetic similarity of P2 and P3. The linkage disequilibrium (LD) was estimated by *r*^*2*^ for the distance classes of < 1 kb in 30 kb distance around the loci *pms3* and *tms5.* The average *r*^*2*^ value reached the threshold of r^2^ < 0.5 at the distance of 3.9 kb for markers around the *tms5* locus while this value remained above threshold for the *pms3* locus (Additional Fig. 5).

**Table 1:** The Fst values for pairwise comparison among populations

	P1	P2	P3	P4	P123
P1	–	0.085	0.149	0.223	–
P2	0.108	–	0.022	0.078	–
P3	0.178	0.045	–	0.085	–
P4	0.252	0.099	0.108	–	0.059
P123	–	–	–	0.078	–

Note: Above diagonal are mean Fst values, while below diagonal are weighted Fst values. The populations (P1-P4) were defined based on the presence of photosensitive male sterility (*pms3*) and temperature sensitive male sterility (*tms5*). P1 has *pms3*, P2 has *tms5*, P3 has both *pms3* and *tms5*, P4 is the conventional breeding lines without male sterility genes, and P123 represents the whole population of genotypes with male sterility genes

### Phylogenetic cluster analysis of the four populations of PTGMS lines of rice

**Fig. 4:**
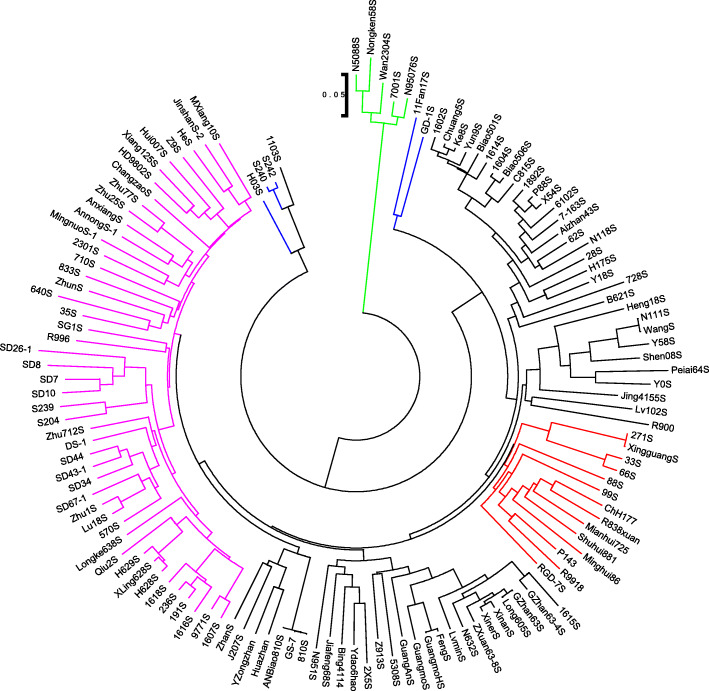
Neighbor joining (NJ) phylogenetic tree of 131 selected genotypes indicating the genetic distances based on SNP markers. The different clusters were indicated in different branch colors. The cluster with green and blue branch color belongs to P1 indicating the root of tree, the cluster with red branch color possessed the conventional breeding lines. The clusters with black and pink branch colors belong to P2 and P3, respectively

The phylogenetic analysis was performed with all selected SNP markers to reveal the ancestral relation among the 118 male sterile lines and 13 conventional breeding lines. According to the neighbor joining (NJ) tree, all the lines could be divided into four clades at the 0.05 genetic distances (Fig. 4). Among them, the first clade consisted of five lines (N5088S, Nongken58S, Wan2304S, 7001S, and N95076S). These lines were at the maximum genetic distance of 0.29 and were the root of the phylogenetic tree. These lines could be the ancestors of other male sterile lines. The second clade was composed of six genotypes, including five genotypes (GD-1S, H03S, S242, S240, and 1103S) belonging to P1, and one genotype (11Fan17S) belonging to P2. Four genotypes (H03S, S242, S240, and 1103S) in this clade grouped together and showed a relatively high distance from other genotypes, while the two other genotypes from P1 (GD1S) and P2 (11Fan17S) made the root of remaining genotypes. These genotypes might be the progenitors of clade 1 lines and the ancestors of the remaining as they showed close relation to other genotypes. The remaining genotypes from P2 and P3 were grouped with the genotypes from P4, indicating their genetic resemblance.

### Principal component analysis of the four populations of PTGMS lines of rice

**Fig. 5:**
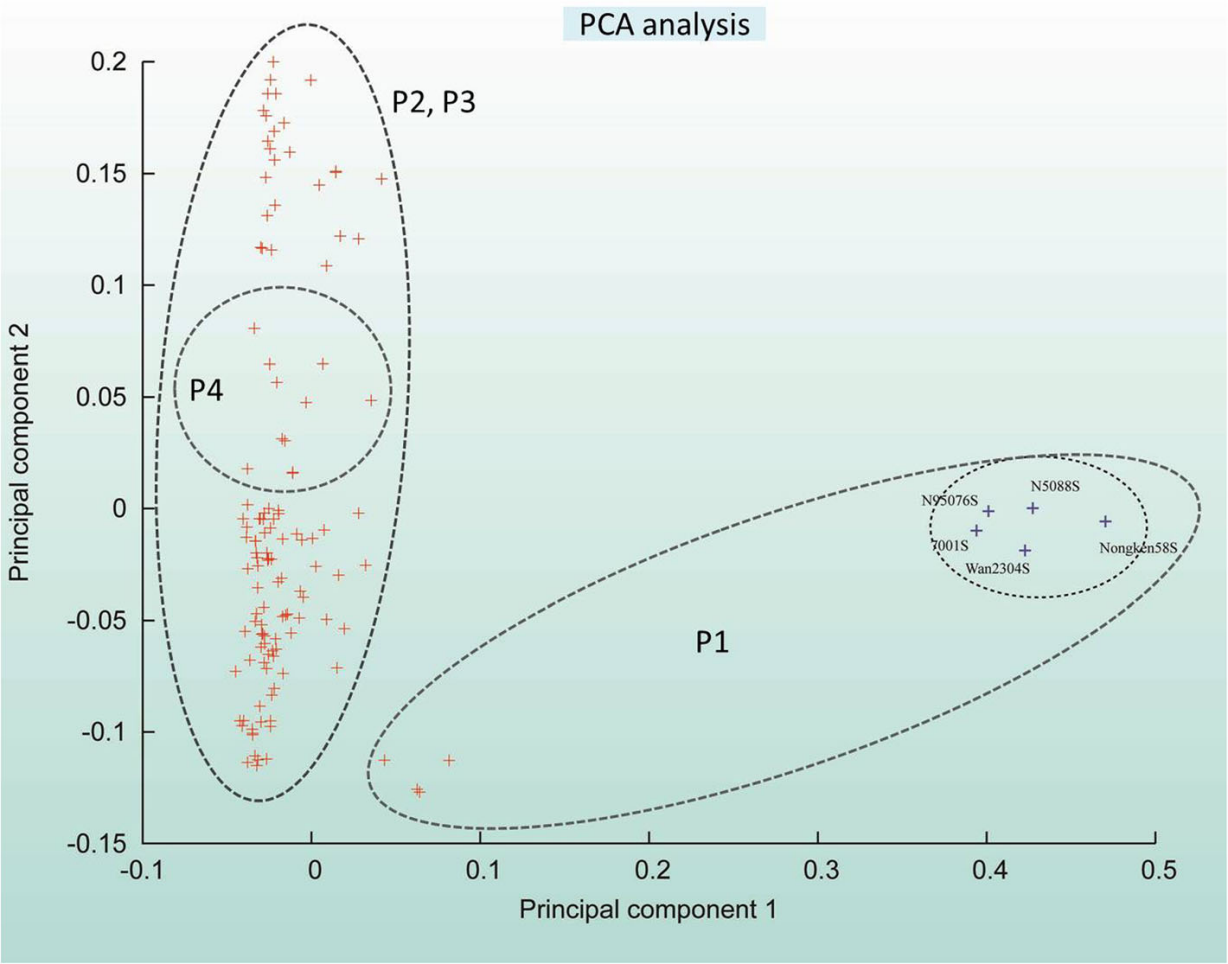
Principal component analysis of 131 rice genotypes, with PC1 and PC2 classifying the whole germplasm into clusters

The results were further supported by the principal component analysis (PCA). The top two principal components PC1 and PC2 explained 19.77 and 7.62% of the total variation, respectively, and divided the germplasm into two major categories. The next two PCs such as PC3 and PC4 explained 6.23 and 3.82% of the total variation, respectively (Additional Fig. 1). One of two clusters grouped the five breeding lines of P1 (clade-I of phylogenetic tree) with a wide genetic distance from other clade, while the other clusters possessed all of the genotypes from P2, P3 and P4 with a narrow genetic distance. Being an independent cluster from others, the Cluster 1 harbored the highest level of genetic differentiation (Table 1, Fig. 5, Additional Fig. 1). Its unique genetic variation pattern could also be evidenced in the top two PCs. The succeeding clusters could further be classified into three overlapping groups (Fig. 5).

### Admixture cluster analysis of the four populations of PTGMS lines of rice

**Fig. 6:**
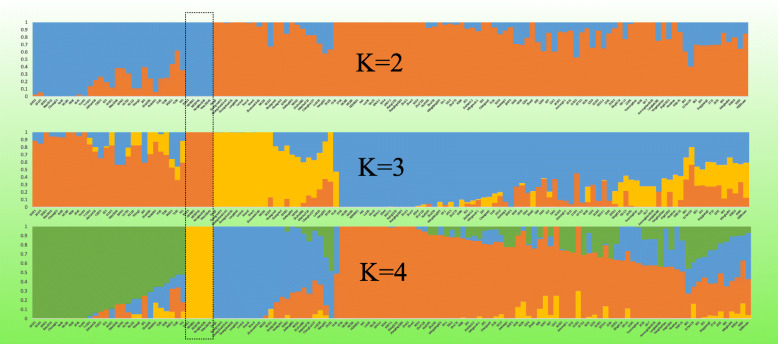
Population admixture analysis of 131 genotypes up to K = 4, indicating the genetically stable and mixed genotypes

To infer the admixture degree across the 131 samples, we further performed an unsupervised admixture analysis with 56 K SNP markers based on K run from 2 to 4. We found that at K = 2, a genetic divergence occurred between the P1 genotypes and their close relatives, while K = 3 and K = 4 sub-divided the groups. At K = 4, the whole germplasm was divided into four (S1, S2, S3, S4) groups of 28, 5, 23 and 75 genotypes, respectively. Except for the genetically distant five genotypes from P1 grouped as S2 in the structure analysis, a potential widespread genetic introgression from conventional breeding lines of P4 to other populations was observed across K = 2 to K = 4 (Fig. 6). There were 10, 10 and 13 genetically pure lines in S1, S3 and S4, and the remaining showed genomic introgression. These results reinforce the previous analysis with pure lines and mixed genomic lines [21, 24]. Among the populations, 5, 31, and 1 genotype from P1, P2, and P4, respectively, were observed to be pure lines. In contrast, all the conventional breeding lines in P4 had the introgressed genomic components. The pure lines specifically the genotypes in S2 are likely the ancestors of the remaining germplasm.

### Genome-wide selective sweep signals, their molecular function and validation

**Fig. 7:**
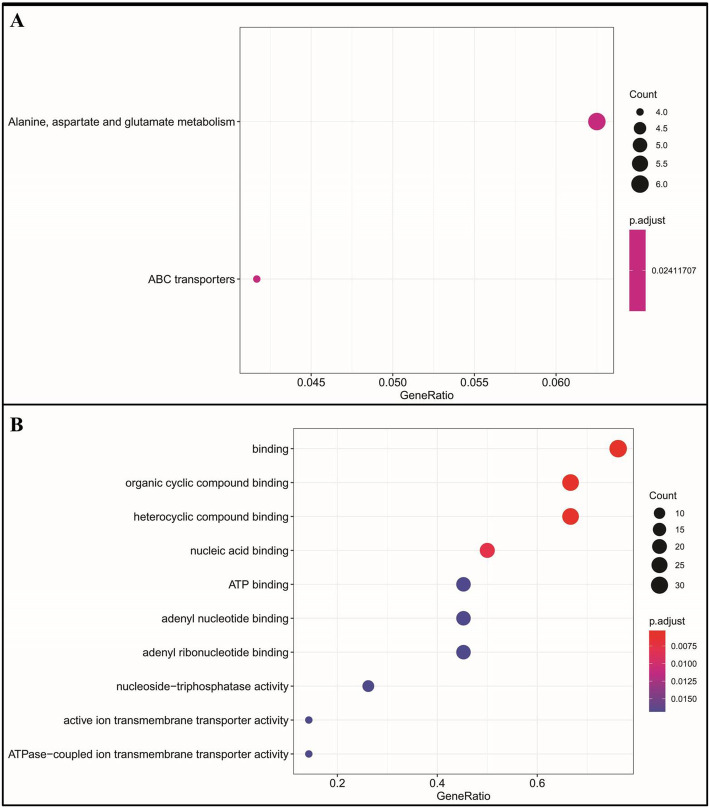
The candidate genes function analysis revealed by (**A**) KEGG and (**b**) (GO databases

In order to better detect genome-wide selection signals related to the male sterility in the genotypes, we divided the populations into male sterile (P1, P2, P3) and male fertile (P4) groups. The high Fst values (top 1%, Fst value> 0.34) were used as criteria for classifying the selective sweeps. There was no selection sweep on chromosome 3, 5, and 8. A total of 1044 candidate genes were found within the sweeps detected on other chromosomes. Some of these genes could be associated with sterility (Additional Table 3). Five sweeps located on chromosomes 1, 2, 4 and 6 exhibited high Fst values (0.888, 0.718, 0.652, 0.650, 0.643) indicating obvious genetic differentiation between male sterile and fertile populations. The largest genomic region of 2 Mb containing 376 candidate genes was observed on chromosome 2 followed by 1 Mb region of chromosome 4 containing 174 candidate genes (Additional Table 2). Kyoto Encyclopedia of genes and genomes (KEGG) pathway enrichment analysis revealed that the candidate genes in the selection sweeps were mainly involved in the ‘Alanine, aspartate and glutamate metabolism’ and ‘ABC transporters’ pathways (Fig. 7A). Gene ontology (GO) analysis revealed 113 GO terms of which the molecular binding was identified as the top enriched ‘Molecular function’ (Fig. 7B).

To further validate the genome-wide selective sweep signals, three pedigree groups A, B, and C were obtained from the rice breeding database according to their breeding history (Additional Fig. 2). In the pedigree groups A, there were 7, 15 and 1 genotypes containing *pms3*, *tms5* and *pms3.tms5* genes, respectively, while the genotypes in group B and C possessed *tms5* gene. The genome wide diversity for all three groups was investigated and the genetic differentiation from conventional genotypes (P4) was studied. A total of 185, 182 and 181 selection sweep signals were observed for pedigree group A, B and C, respectively, in comparison with conventional lines at the Fst threshold of top 5% selective sweep signals (Additional Fig. 3, Additional Table 4). Among the top 1% selective sweeps, we found the same genomic regions as identified in top genetic differentiation hits for male sterile (P1, P2, and P3) and conventional breeding lines (P4). The genes in candidate regions were subjected to GO analysis. The ‘Binding’ type of molecular functions involved in ‘metabolic’ and ‘signal transduction’ processes in ‘Nucleus’ and ‘membrane complexes’ were on top hits (Additional Fig. 4).

### Selection of core germplasm

All the genotypes were arranged on the basis of genetic diversity and top 30% genotypes were selected at the first step. All the cluster analysis and the male sterility allele’s evaluation grouped the germplasm into four groups. The genotypes in each group were arranged on the basis of their available sterility allele and the genetic diversity among the genotypes. The top 10% of commonly selected genotypes from both procedures resulted in the selection of 13 genotypes to develop a core collection. Among them, 2 (GD1S and N5088S), and 11 (ZhunS, Biao506S, Shen08S, JinShanS, 2301S, S204, Ke8S, Long605S, 66S, Longke638S, and 99S) genotypes were selected from P1 and P2, respectively. Furthermore, one genotype (S239) from P3 and one conventional genotype (Shuhui881) were also included in the core collection.

## Discussion

Crop breeding programs aim to harness genetic diversity for desirable phenotypes to meet human demands [25]. To achieve the ideal genotype, the bottleneck effect on phenotypic selection in elite varieties during rice breeding programs have dramatically narrowed down their genetic diversity [26]. However, the information about genes which generated the changes in desirable phenotypes in elite rice varieties is limited. Even some genes may cause the transition of PGMS line to TGMS line [27]. The photosensitive (*pms3*) and temperature sensitive male sterility (*tms5*) genes could classify the collection of 131 two-line male sterile lines into four major populations in this study. The information about available PTGMS genes and markers will not only help the direct selection of genotypes in hybrid breeding but also for the future research programs. Based on these genetic markers, other germplasm resources could also be evaluated and manipulated to enhance the genetic diversity. In addition, genome-wide marker analysis of various rice populations has demonstrated that shifts in genetic population structures have occurred multiple times in history [28]. The shift of genetic diversity in the local gene pool may be managed with the use of germplasm for human demands in rice breeding programs [28].

### Genetic purity

Maintenance of genetic purity in inbred lines by minimizing residual heterozygosity (heterogeneity) is important for quality seed production [29]. The threshold value may vary depending on the purpose of the line development program and level of inbreeding. In the current study, the genome of all four populations revealed a high level of homozygosity with 93 to 97% homozygous alleles (Fig. 2, Additional Table 2). Generally, the male sterile lines are bred by backcross for many generations. Therefore, they may show morphological differences but have minor to negligible differences in the nuclear genome. This level of heterogeneity may be due to the use of different methods for line-maintenance or the natural variation through crossing over. Currently, there is more demand in developing uniform hybrids using genetically pure parental lines, especially doubled haploid lines [29]. As a result, rice breeders are using fixed lines in their new pedigree starts up and advance each generation through selfing than sib-mating. In the long-term solution to improve the homogeneity, it is recommended to use doubled haploid (DH) technology in developing genetically pure DH lines that can be derived in a short period of time [29].

### Genome wide nucleotide diversity

There was a low nucleotide diversity (π) in all four populations (Fig. 2, Additional Table 2), which indicated a close relation among the genotypes and suggested the limited number of available signature-genes in the germplasm. The lowest genetic diversity in P4 revealed that the conventional breeding lines are facing an intensive selection pressure than the PTGMS lines. The widest range of genetic diversity was observed for P3, while it was the narrowest in P2. Among the male sterile populations, P2 and P3 were selected at similar levels and showed higher selective sweep than P1. In general, the average genetic diversity value of 0.67 and 0.90 within Asian cultivated rice and common wild rice has been observed with SSR markers [30]. The analysis of 4408 accessions of Chinese cultivated rice germplasm with 12 isozyme loci reported an average gene diversity range from 0.012 to 0.547 [31]. In our experiment, the results showed that the genetic diversity of the tested materials was very low. It indicates the genetic similarity among the genotypes and a similar pedigree, resulting in the decreased nuclear genome diversity.

### Genetic relationship and population structure among the two-line hybrid rice lines

To understand the genetic admixture, the relative kinship coefficients are used as indicators of genetic relationship among the pairs of genotypes. The values of admixture ranged from zero for lack of relation to higher values for stronger relationships. The results from admixture and PCA analyses in this study revealed that the genetic divergence first occurred between the P1 genotypes and their close relatives. The presence of pure lines in each subgroup reinforced the population admixture. The admixture results were reinforced by the phylogenetic analysis and revealed the ancestral relationship among the 131 lines of rice (Fig. 4). As a whole, the majority of the genotypes could not be well grouped into specific cluster as indicated by the phylogenetic and admixture analyses, suggestive of a complex genetic structure. Besides, it was hard to separate the rice breeding lines of different populations from each other, indicating high admixture among them.

### Genome-wide genetic differentiation

The accumulation of differences in allelic frequencies between completely or partially isolated populations due to evolutionary forces such as selection or genetic drift could be evaluated by population differentiation analysis. The population differentiation was studied by Fst-values. The P1 population was genetically differentiated from other populations. There was no significant difference between population P3 from P4 and the P1 from P2. It suggests a driving force of *pms3* and *tms5* genes in shaping the genetic variation pattern, and indicates the genetic similarity of P2 with P3. The results may not totally support the arguments given by [32, 33] that a critical role might have been played by distance based isolation in shaping the genomic variation. Hence a strong artificial selection can be proposed as the main driving force in shaping rice genomic variation.

### Genome-wide selective sweep signals and the role of candidate genes

The genome-wide selection signals related to the male sterility in genotypes was also observed by comparing the male sterile (P1, P2, P3) and male fertile (P4) groups. There are various traits in plants such as plant height, seed color, and stem angle that can be influenced by selective sweep [29, 34]. The study on distinct phenotypic evaluation of rice revealed the physiological and morphological effects of selection sweep on rice breeding [29, 34]. The candidate genes identified in this study can be functionally characterized for their roles in genetic differentiation and induction of male sterility.

As shown in the Manhattan plot (Fig. 3, Additional Table 3), the highest Fst score (0.888) was observed within 120 kb interval on chromosome 4 (4:7920001–8,040,000). This region harbored 19 protein-coding genes, including (i) RPM1 disease resistance protein which facilitates a rapid and sustained increase in cytosolic calcium that is necessary for the oxidative burst and hypersensitive cell death ([35], (ii) TRAF-type zinc finger family protein, its function is extraordinarily diverse and includes DNA recognition, RNA packaging, transcriptional activation, regulation of apoptosis, protein folding and assembly, and lipid binding [36], (iii) wall associated receptor like Kinase (*osWAK*) protein, its central role in resisting a range of fungal and bacterial diseases [37].

Two of the selective sweeps on chromosome 2 (2:4860001–5,080,000 & 6,080,001–6,600,000) consisted of 48 and 103 candidate genes, respectively. Amon the candidate genes, we have (i) AP2 domain containing genes which are necessary for flower development, stem cell maintenance, seed development, and abiotic stresses resistance [38, 39], (ii) MYB protein, which are the key factors in the regulatory networks controlling development, metabolism and responses to biotic and abiotic stresses [40], (iii) GDSL like lipase, which possesses anti-microbial activity and regulates pathogen resistance in association with ethylene signaling [41], (iv) F-box domain (*osFBX39*), which was reported to contribute in regulation of drought tolerance and regulation of stomatal closure in plants [42] and multi drug resistance proteins. The candidate genes mentioned above could be potentially influencing traits of adaptation in rice.

The selection sweep results were further validated by estimation of genetic differentiation between conventional breeding lines in P4 and the three pedigree groups (A, B, C) based on their breeding history. The ‘Nongkan58S’ with *pms3*, and ‘Annong S-1’ and ‘Zhu1S’ with *tms5* genes were observed as the root ancestors of all the other genotypes in groups A, B, and C, respectively (Additional Fig. 2). Annong S-1 has been reported as the first *indica* TGMS line [5]. Its significant reaction to temperature and light in fertility expression, inheritance of sterility and physio-biochemical characteristics have also been reported [5]. Most of the PTGMS lines in the breeding panel for two-line rice hybrid system in the study possessed the *tms5* gene, which may result from the major contribution of Annong S-1 to their development.

### Selection of core germplasm

With the identification of genetic similarity and diversity among the PTGMS lines, it was important to design a genetically diverse sample with all possible genetic variation among the population. Selecting a representative sample of all the diversity in the large collection would facilitate the enhanced use of germplasm in the breeding programs [43]. The selection of core collection from two-line PTGMS lines will help accelerate the work on germplasm evaluation and development of new breeding plan. The open sharing of PTGMS resources will also be helpful to minimize the adverse environmental effects on seed production in two-line hybrids [44]. Such samples are favored for excellent exploitation and utilization of diversity, and would be cost effective and easy to maintain by individual breeders [43]. A core collection is a subset, consisting of ~ 10% of total genotypes, which between them capture most of the available diversity in the entire collection and nearly as diverse as the entire collection [24]. Hence, the core PTGMS collection designed in this study carrying the maximum possible diversity from all four populations will result in more efficient and effective utilization of two-line PTGMS germplasm in rice. This core collection should be updated periodically with the availability of new PTGMS lines and other biological information. The information of the genetic diversity among the PTGMS lines will help to select the suitable parental lines for sustainable hybrid rice seed production and also for the evaluation of various quantitative and qualitative traits of agronomic importance.

## Conclusion

In the present study, the diversity, relationships, selection sweep and breeding inferences were investigated in a collection of 131 rice genotypes including, 118 male sterile lines and 13 conventional breeding lines. The collection was clearly grouped into 4 gene pools according to the presence of the male sterility genes. Based on the within and among populations genetic diversity, a core collection was constructed which will be useful for future breeding efforts. The population containing the photosensitive gene (*pms3*) was highly divergent and suggested to be the ancestor of other populations. Several candidate genes were identified in the selective sweeps and could the targets of future studies to unravel their contribution to rice adaptation, genetic differentiation and male sterility.

## Methods

### Plant materials and genotyping

To gain insight into the genetic relationship of the male-sterile germplasm and to better utilization in rice breeding programs, we employed 131 genotypes including 118 2-line PTGMS rice lines and 13 conventional breeding lines (Additional Table 1). The germplasm was obtained from Hunan Hybrid Rice Research Center, Hunan China, and grown in the experimental farm of the same institute. All the standard cultivation practices were adopted. The fresh leaves of at least six plants of each male sterile line were collected and total DNA was extracted by the simple CTAB method with minor modifications [45]. The DNA samples (50–100 ng/μL per sample) in a 96-well plate format were prepared for genotyping with high-density Illumina 56 K infinium SNP Chip in Huazhi Biotech Co. Ltd., Changsha, China. Monomorphic markers, with missing values < 20%, with a minor allele frequency < 5%, and/or showing unclear SNPs were excluded from the analysis. The filtered genotypic data was used in subsequent analysis.

### PCR reaction and genomic screening

Two male sterile genes, *pms3* and *tms5* were used to characterize the germplasm. The polymerase chain reaction (PCR) was conducted in 20 μL containing 3 μL primers, 2 μL 10 × PCR buffer (involving 15 mmol/L MgCl_2_), 0.3 μL dNTPs (10 mmol/L), 50–100 ng template DNA, 1 U *Taq* enzyme. The reaction program was pre-denatured at 94 °C for 5 min, 35 cycles with 1 min at 94 °C, 1 min at 55 °C and 1 min at 72 °C, finally extended for 5 min at 72 °C. Then, the amplified products were electrophoresed on a 6% denatured polyacrylamide gel. On the basis of male sterility genes the germplasm was clustered into four classes as the genotypes carrying “*pms3*”, “*tms5*”, both “*pms3* and *tms5*” genes, and the conventional breeding strains without any male sterility allele (Additional Table 1).

### Statistical analysis

To investigate the relationship among genotypes, the population structure was calculated by using all of filtered SNPs in model-based program Structure v2.4.2 [46]. Ten independent simulations were carried out for each K (the number of populations) ranging from 1 to 6. For each simulation, 10,000 iterations before a burn-in length of 50,000 Markov Chain Monte Carlo replications were performed with the selection of admixture and related frequency models. The LnP(D) values and optimal K-value was estimated using Evanno’s 1 K method [46] with online tool Structure Harvester [47]. Furthermore, principal component analysis was performed. Genome-wide diversity (π) within each population and the pair wise genetic differentiation between each population was computed using VCFtools version 0.1.14 [48], with a window size of 100 kb and a step size of 20 kb [49]. The linkage disequilibrium decay rate was estimated by r^2^ values with distance across the loci. The < 1 kb distance window for SNP pairs in 30 kb distance was used. The NJ method based on Nei’s genetic distances among genotypes [50] using the Mega X [51] followed for phylogenetic cluster analysis of germplasm. The tree was visualized and edited by Evolview online tool [52]. PowerMarker v3.25 [53] was used to identify the core germplasm for breeding. The PTGMS genes based clusters of the germplasm were compared in seven possible combinations viz.; 1) ‘*pms3’* vs ‘*tms5’, 2) ‘pms3’* vs *‘pms3 + tms5’, 3) ‘pms3’* vs *Normal, 4)* ‘*tms5’* vs *‘pms3 + tms5’, 5) ‘tms5’* vs *Normal, 6) ‘pms3 + tms5’* vs *Normal, and 7)’ pms3,tms5,pms3 + tms5’* vs normal. The results from comparison of normal genotypes to others (PTGMS genes containing) lines were used to identify the candidate genes in selection sweeps and for their GO [54] and KEGG enrichment analyses [55]. Finally, the top 10% of germplasm repeatedly discovered in both methods was identified as the core germplasm for future rice breeding.

## Supplementary Information

**Additional file 1: Figure 1**. Principal component analysis of 131 rice genotypes, with PC1, PC2 PC3 and PC4 classifying the whole germplasm into clusters. **Figure 2**. The pedigree groups A, B and C obtained from the rice breeding database according to their breeding history, the top one is the ancestor of the other below in the group. The available male sterile gene is indicated in braces with the name of each ancestral genotype. **Figure 3**. Top 1% selection sweeps on nine chromosomes indicating the genetic differentiation evaluated by Fst values among four populations, the red box in the Chromosome 2 indicated commonly identified and validated top selective sweep. **Figure 4**. The top enriched gene ontological function revealed Gene Ontology data bases for candidate genes from selective sweep analysis of conventional breeding lines versus pedigree group A (A), B (B) and C (C).

**Additional file 2: Figure 5**. Linkage disequilibrium decay plot, the pairwise LD values (r2) plotted against the physical distance.

**Additional file 3: Table 1**. The list of 118 two-line hybrid environment sensitive genetically male sterile (ESGMS) lines of rice.

**Additional file 4: Table 2**. Chromosome wise values of various genetic diversity parameters for four populations.

**Additional file 5: Table 3**. Top 1% selection sweeps on nine chromosomes indicating the genetic differentiation evaluated by Fst values among four populations.

**Additional file 6: Table 4**. The genome wide selection sweeps indicating the genetic differentiation between the conventional breeding lines and the pedigree groups A, B, and C evaluated by Fst values at the threshold of top 5%.

## Data Availability

Plant materials can be obtained at Hunan Hybrid Rice Research Center, China. DNA sequencing data are deposited at DDBJ/ENA/GenBank under the accession number: QWGC02800000. Data will be publicly released upon publication of this manuscript.

## Abbreviations

CMS: Cytoplasmic Male Sterile
DH: Double Haploid
EGMS: Environmentally sensitive Genic Male Sterile
GO: Gene Ontology
KEGG: Kyoto Encyclopedia of Genes and Genomes
MAS: Marker Assisted Selection
NGS: Next Generation Sequencing
NJ: neighbor joining
PCA: Principal Component Analysis
PCR: Polymerase Chain Reaction
PTGMS: Photoperiod- and Thermo-sensitive Genic sensitive Male Sterile
QTL: Quantitative Trait Locus
SNP: Single Nucleotide Polymorphism
TGMS: Temperature sensitive Genic Male Sterile

## References

[1] Mehrotra R, Bhalothia P, Bansal P, Basantani MK, Bharti V, Mehrotra S. Abscisic acid and abiotic stress tolerance–Different tiers of regulation. J Plant Physiol. 2014;171(7):486–96. 10.1016/j.jplph.2013.12.007 24655384

[2] Cheng S-H, Zhuang J-Y, Fan Y-Y, Du J-H, Cao L-Y. Progress in research and development on hybrid rice: a super-domesticate in China. Ann Bot. 2007;100(5):959–66. 10.1093/aob/mcm121 PMC2759200 17704538

[3] Ullah A, Sun H, Yang X, Zhang X (2017). Drought coping strategies in cotton: increased crop per drop. Plant Biotechnol J.

[4] Mengchen Z, Shan W, Jianfang Y, Shuiyong S, Xin X, Qun X, Xiaoping Y, Xinghua W, Yaolong Y (2019). Classification and identification of indica P/TGMS lines in China. Rice Sci.

[5] Deng H, Shu F, Yuan D (1999). An overview of research and utilization of Annong S-1 (in Chinese). Hybrid Rice.

[6] Singh S, Singh P, Singh DK, Singh AK. Hybrid rice development: two line and three line system. J BIOLOGIX. 2013;178:178-95.

[7] Xu J, Wang B, Wu Y, Du P, Wang J, Wang M, Yi C, Gu M, Liang G (2011). Fine mapping and candidate gene analysis of ptgms2-1, the photoperiod-thermo-sensitive genic male sterile gene in rice (Oryza sativa L.). J Theoretical Appl Genetics.

[8] J-q L, Cai S-X, J-h F, Wei L, Cheng G-P (2007). Comparisons on genetic diversity among the Isonuclear-Alloplasmic male sterile lines and their maintainer lines in Rice. J Rice Sc.

[9] Rout D, Jena D, Singh V, Ahlavat MK, Arsode P, Singh P, et al. Hybrid Rice research: current status and prospects. In: Recent Advances in Rice Research: IntechOpen; 2020.

[10] Wang W, Mauleon R, Hu Z, Chebotarov D, Tai S, Wu Z, Li M, Zheng T, Fuentes RR, Zhang F (2018). Genomic variation in 3,010 diverse accessions of Asian cultivated rice. J Nat.

[11] Marulanda JJ, Mi X, Melchinger AE, Xu J-L, Würschum T, Longin CFH (2016). Optimum breeding strategies using genomic selection for hybrid breeding in wheat, maize, rye, barley, rice and triticale. J Theoretical Appl Genetics.

[12] Li J-Y, Wang J, Zeigler RS (2014). The 3,000 rice genomes project: new opportunities and challenges for future rice research. J Gigascience.

[13] Rashid MAR, Zhao Y, Zhang H, Li J, Li Z (2016). Nucleotide diversity, natural variation, and evolution of flexible culm-1 and strong culm-2 lodging resistance genes in rice. Genome.

[14] Zhao Y, Zhao W, Jiang C, Wang X, Xiong H, Todorovska EG, Yin Z, Chen Y, Wang X, Xie J (2018). Genetic architecture and candidate genes for deep-sowing tolerance in rice revealed by non-syn GWAS. Front Plant Sci.

[15] Yu J, Xiong H, Zhu X, Zhang H, Li H, Miao J, Wang W, Tang Z, Zhang Z, Yao G (2017). OsLG3 contributing to rice grain length and yield was mined by ho-LAMap. BMC Biol.

[16] Morales KY, Singh N, Perez FA, Ignacio JC, Thapa R, Arbelaez JD, Tabien RE, Famoso A, Wang DR, Septiningsih EM, Shi Y, Kretzschmar T, McCouch SR, Thomson MJ (2020). An improved 7K SNP array, the C7AIR, provides a wealth of validated SNP markers for rice breeding and genetics studies. PLoS One.

[17] Singh S, Mahato AK, Jayaswal PK, Singh N, Dheer M, Goel P, Raje RS, Yasin JK, Sreevathsa R, Rai V (2020). A 62K genic-SNP chip array for genetic studies and breeding applications in pigeonpea (*Cajanus cajan* L. Millsp.). Sci Rep.

[18] Singh N, Jayaswal PK, Panda K, Mandal P, Kumar V, Singh B, Mishra S, Singh Y, Singh R, Rai V (2015). Single-copy gene based 50 K SNP chip for genetic studies and molecular breeding in rice. Sci Rep.

[19] Thomson MJ, Singh N, Dwiyanti MS, Wang DR, Wright MH, Perez FA, DeClerck G, Chin JH, Malitic-Layaoen GA, Juanillas VM (2017). Large-scale deployment of a rice 6 K SNP array for genetics and breeding applications. Rice (New York, NY).

[20] Yu H, Xie W, Li J, Zhou F, Zhang Q (2014). A whole-genome SNP array (RICE6K) for genomic breeding in RICE. Plant Biotechnol J.

[21] Zhao K, Tung CW, Eizenga GC, Wright MH, Ali ML, Price AH, Norton GJ, Islam MR, Reynolds A, Mezey J, McClung AM, Bustamante CD, McCouch SR (2011). Genome-wide association mapping reveals a rich genetic architecture of complex traits in Oryza sativa. Nat Commun.

[22] McCouch SR, Wright MH, Tung CW, Maron LG, McNally KL, Fitzgerald M, Singh N, DeClerck G, Agosto-Perez F, Korniliev P (2016). Open access resources for genome-wide association mapping in rice. Nat Commun.

[23] Singh N, Jayaswal PK, Panda K, Mandal P, Kumar V, Singh B, Mishra S, Singh Y, Singh R, Rai V (2015). Single-copy gene based 50 K SNP chip for genetic studies and molecular breeding in rice. Sci Rep.

[24] Zhang H, Zhang D, Wang M, Sun J, Qi Y, Li J, Wei X, Han L, Qiu Z, Tang S, Li Z (2011). A core collection and mini core collection of Oryza sativa L. in China. TAG Theoretical and applied genetics Theoretische und angewandte Genetik.

[25] Louwaars NP (2018). Plant breeding and diversity: a troubled relationship?. J Euphytica.

[26] Fujino K, Hirayama Y, Kaji R (2019). Marker-assisted selection in rice breeding programs in Hokkaido. J Breed Sci.

[27] Zhang H-L, Chen X-Y, Huang J-Z, EZ-G, Gong J-Y, Shu Q-Y (2015). Identification and transition analysis of photo- /Thermo-sensitive genic male sterile genes in two-line hybrid Rice in China (Chinese paper with English abstract). Sci Agric Sin.

[28] Shinada H, Yamamoto T, Yamamoto E, Hori K, Yonemaru J, Matsuba S, Fujino K (2014). Historical changes in population structure during rice breeding programs in the northern limits of rice cultivation. J Theoretical Appl Genetics.

[29] Wegary D, Teklewold A, Prasanna BM, Ertiro BT, Alachiotis N, Negera D, Awas G, Abakemal D, Ogugo V, Gowda M, Semagn K (2019). Molecular diversity and selective sweeps in maize inbred lines adapted to African highlands. Sci Rep.

[30] Beyene Y, Botha A, Myburg AA (2006). Genetic diversity among traditional Ethiopian Highland maize accessions assessed by simple sequence repeat (SSR) markers. Genet Resour Crop Evol.

[31] Beyene Y, Botha A, Myburg AA (2006). Genetic diversity in traditional Ethiopian highland maize accessions assessed by AFLP markers and morphological traits. Biodivers Conserv.

[32] Chen L, Wang X, Cheng D, Chen K, Fan Y, Wu G, You J, Liu S, Mao H, Ren J (2019). Population genetic analyses of seven Chinese indigenous chicken breeds in a context of global breeds. J Anim Genetics.

[33] Nie C, Almeida P, Jia Y, Bao H, Ning Z, Qu L (2019). Genome-wide single-nucleotide polymorphism data unveil admixture of Chinese indigenous chicken breeds with commercial breeds. J Genome Biol Evol.

[34] Yuan Y, Zhang Q, Zeng S, Gu L, Si W, Zhang X, Tian D, Yang S, Wang L (2017). Selective sweep with significant positive selection serves as the driving force for the differentiation of japonica and indica rice cultivars. BMC Genomics.

[35] Grant M, Brown I, Adams S, Knight M, Ainslie A, Mansfield J (2000). The RPM1 plant disease resistance gene facilitates a rapid and sustained increase in cytosolic calcium that is necessary for the oxidative burst and hypersensitive cell death. Plant J.

[36] Laity JH, Lee BM, Wright PE (2001). Zinc finger proteins: new insights into structural and functional diversity. Curr Opin Struct Biol.

[37] Li H, Zhou SY, Zhao WS, Su SC, Peng YL (2009). A novel wall-associated receptor-like protein kinase gene, OsWAK1, plays important roles in rice blast disease resistance. Plant Mol Biol.

[38] Xie Y, Yu X, Jiang S, Xiao K, Wang Y, Li L, Wang F, He W, Cai Q, Xie H, Zhang J (2020). OsGL6, a conserved AP2 domain protein, promotes leaf trichome initiation in rice. Biochem Biophys Res Commun.

[39] Xie Z, Nolan TM, Jiang H, Yin Y (2019). AP2/ERF transcription factor regulatory networks in hormone and abiotic stress responses in Arabidopsis. Front Plant Sci.

[40] Dubos C, Stracke R, Grotewold E, Weisshaar B, Martin C, Lepiniec L (2010). MYB transcription factors in Arabidopsis. Trends Plant Sci.

[41] Lee DS, Kim BK, Kwon SJ, Jin HC, Park OK (2009). Arabidopsis GDSL lipase 2 plays a role in pathogen defense via negative regulation of auxin signaling. Biochem Biophys Res Commun.

[42] An J, Li Q, Yang J, Zhang G, Zhao Z, Wu Y, Wang Y, Wang W (2019). Wheat F-box protein TaFBA1 positively regulates plant drought tolerance but negatively regulates stomatal closure. Front Plant Sci.

[43] Zhang Y, Zhang X, Che Z, Wang L, Wei W, Li D (2012). Genetic diversity assessment of sesame core collection in China by phenotype and molecular markers and extraction of a mini-core collection. BMC Genet.

[44] SI H-M, Fu Y-P, Liu W-Z, Sun Z-X, Hu G-C (2012). Pedigree analysis of photoperiod-thermo sensitive genic male sterile Rice (Chinese paper with English abstract). Acta Agron Sin.

[45] Eslami G, Khalatbari-Limaki S, Ehrampoush MH, Gholamrezaei M, Hajimohammadi B, Oryan A (2017). Comparison of three different DNA extraction methods for Linguatula serrata as a food born pathogen. J Iran J Parasitol.

[46] Evanno G, Regnaut S, Goudet J (2005). Detecting the number of clusters of individuals using the software STRUCTURE: a simulation study. Mol Ecol.

[47] Earl DA (2012). STRUCTURE HARVESTER: a website and program for visualizing STRUCTURE output and implementing the Evanno method. Conserv Genet Resour.

[48] Danecek P, Auton A, Abecasis G, Albers CA, Banks E, DePristo MA, Handsaker RE, Lunter G, Marth GT, Sherry ST (2011). The variant call format and VCFtools. J Bioinformatics.

[49] Weir BS, Cockerham CC. Estimating F-statistics for the analysis of population structure. J evolution. 1984;38:1358–70. 10.1111/j.1558-5646.1984.tb05657.x 28563791

[50] Nei M (1978). Estimation of average heterozygosity and genetic distance from a small number of individuals. Genetics.

[51] Kumar S, Stecher G, Li M, Knyaz C, Tamura K (2018). MEGA X: molecular evolutionary genetics analysis across computing platforms. Mol Biol Evol.

[52] Subramanian B, Gao S, Lercher MJ, Hu S, Chen W-H (2019). Evolview v3: a webserver for visualization, annotation, and management of phylogenetic trees. J Nucleic Acids Res.

[53] Liu K, Muse S. PowerMarker: integrated analysis environment for genetic diversity in core collection accessions of wild barley, Hordeum vulgare ssp. spontaneum. Hereditas. 2005;136:67–73. 10.1034/j.1601-5223.2002.1360110.x 12184491

[54] Harris MA, Clark J, Ireland A, Lomax J, Ashburner M, Foulger R, Eilbeck K, Lewis S, Marshall B, Mungall C, Richter J, Rubin GM, Blake JA, Bult C, Dolan M, Drabkin H, Eppig JT, Hill DP, Ni L, Ringwald M, Balakrishnan R, Cherry JM, Christie KR, Costanzo MC, Dwight SS, Engel S, Fisk DG, Hirschman JE, Hong EL, Nash RS, Sethuraman A, Theesfeld CL, Botstein D, Dolinski K, Feierbach B, Berardini T, Mundodi S, Rhee SY, Apweiler R, Barrell D, Camon E, Dimmer E, Lee V, Chisholm R, Gaudet P, Kibbe W, Kishore R, Schwarz EM, Sternberg P, Gwinn M, Hannick L, Wortman J, Berriman M, Wood V, de la Cruz N, Tonellato P, Jaiswal P, Seigfried T, White R, Gene Ontology Consortium (2004). The gene ontology (GO) database and informatics resource. Nucleic Acids Res.

[55] Kanehisa M, Sato Y, Kawashima M, Furumichi M, Tanabe M (2016). KEGG as a reference resource for gene and protein annotation. Nucleic Acids Res.

