# Supplementary material for: Genetic relationships and identification of core germplasm among rice photoperiod- and thermo-sensitive genic male sterile lines
Source: BMC Plant Biol. 2021 Jul 2;21:313. doi: 10.1186/s12870-021-03062-x (PMC8252326; doi:10.1186/s12870-021-03062-x)

**Genetic relationships and identification of core germplasm among photoperiod- and thermo-sensitive genic male sterile rice varieties**

Xianwen Zhang^1,2,3^, Qiang He^2^, Wuhan Zhang^2^, Fu Shu^2^, Weiping Wang^2^, Zhizhou He^3^, Hairong Xiong^4^, Junhua Peng^3^, Huafeng Deng^2*^

1. College of Bioscience and Biotechnology, Hunan Agricultural University, 410128, Changsha, China.

2. State Key Laboratory of Hybrid Rice, Hunan Hybrid Rice Research Center, Hunan Academy of Agricultural Sciences, 410125, Changsha, China.

3. Huazhi Biotech Co. Ltd, 410125, Changsha, China.

4. School of Chemistry and Materials Science, Hunan Agricultural University, 410128, Changsha, China.

**Additional files**

**Additional Figure 1:** Principal component analysis (PCA) of 131 rice genotypes, with PC1, PC2 PC3 and PC4 classify the whole germplasm into clusters.


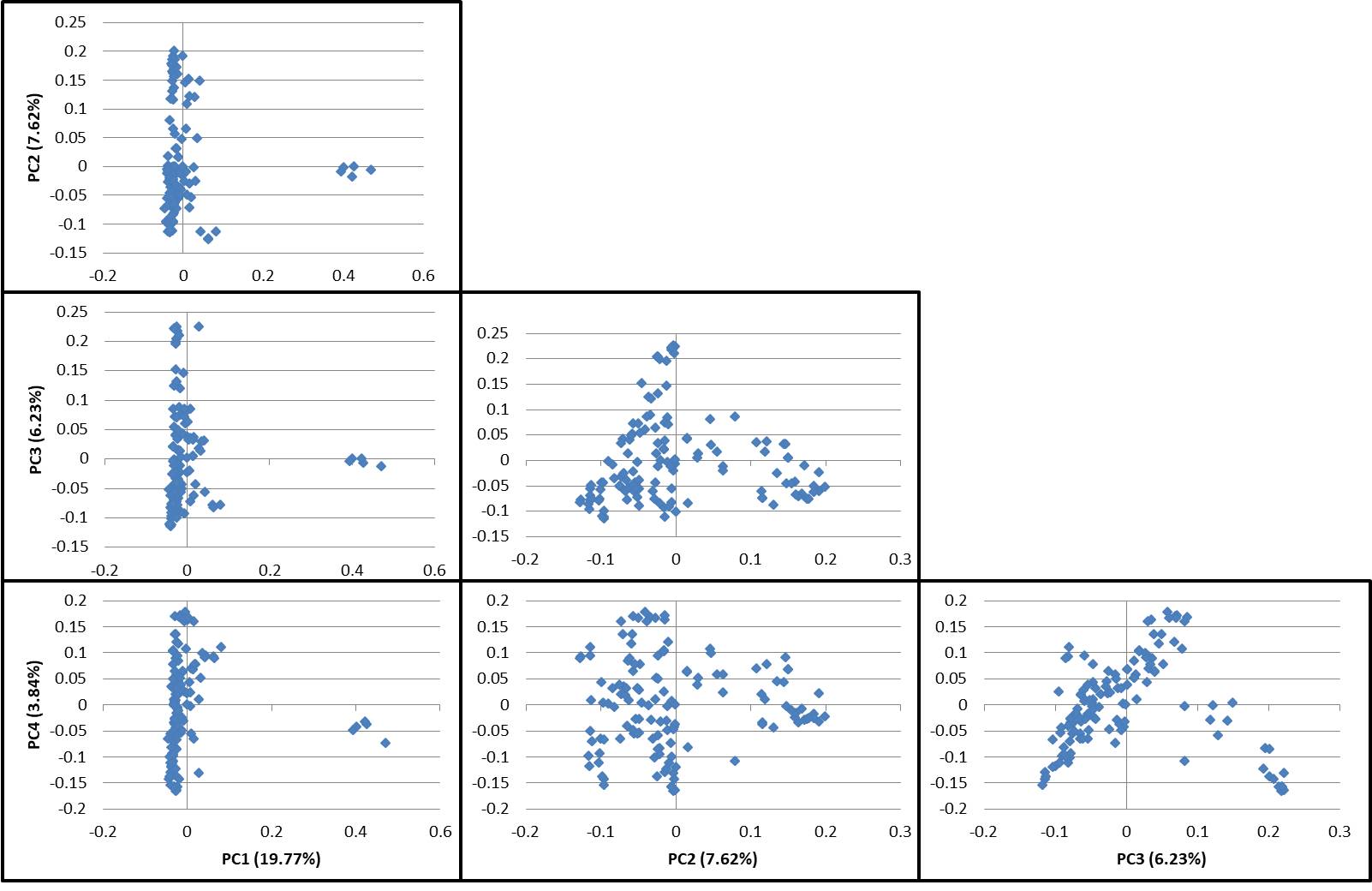


**Additional Figure 2:** The pedigree groups A, B and C obtained from the rice breeding database according to their breeding history, the top one is the ancestor of the other below in the group. The available male sterile gene is indicated in braces with the name of each ancestral genotype.


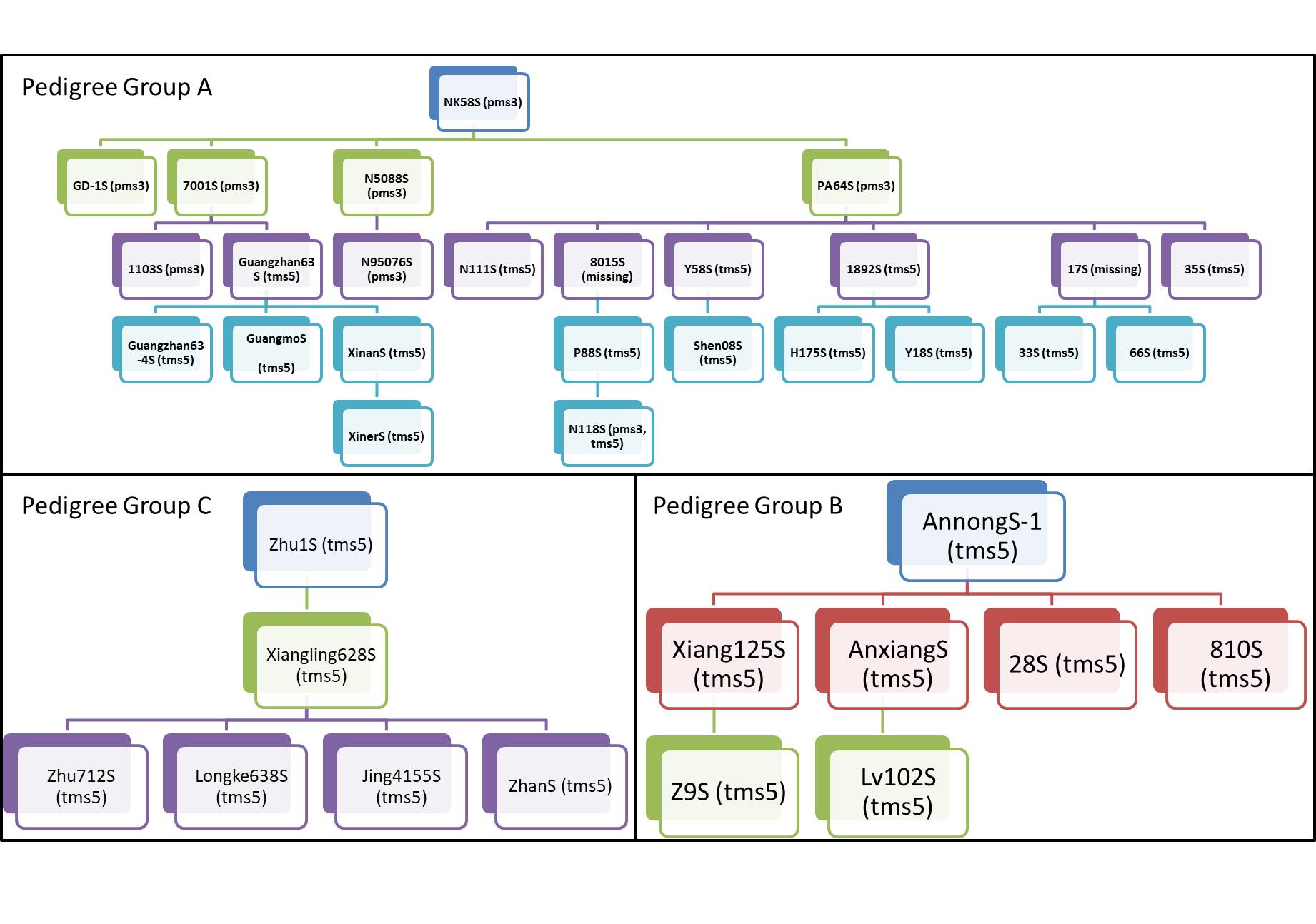


**Additional Figure 3:** Top 1% selection sweeps on nine chromosomes indicating the genetic differentiation evaluated by FST values among four populations, the red box in Chromosome 2 indicated commonly identified and validated top selective sweep.


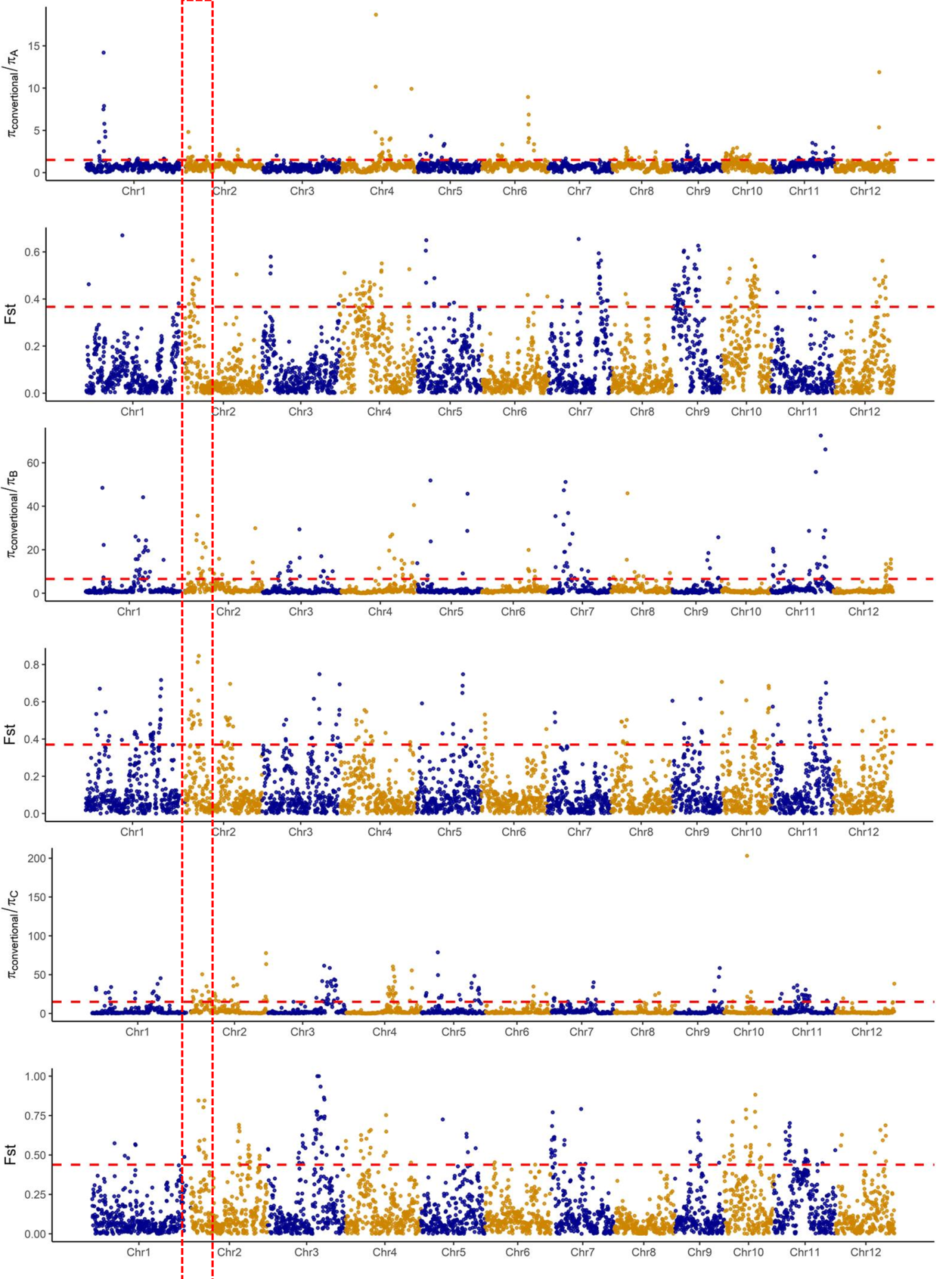


**Additional Figure 4:** The top enriched gene ontological function revealed Gene Ontology (GO) data bases for candidate genes from selective sweep analysis of conventional breeding lines versus pedigree group A (A), B (B) and C(C).


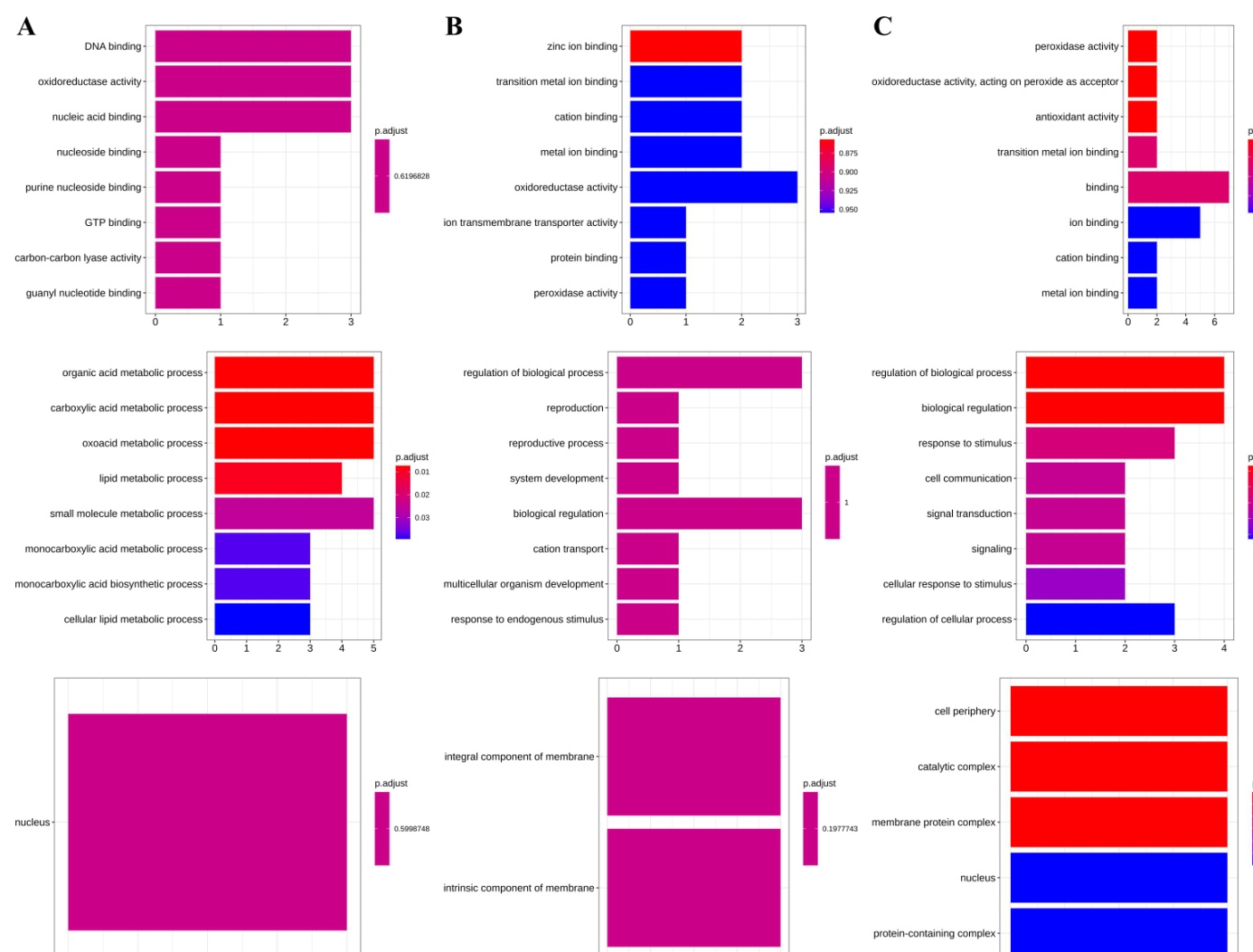

Supplement: Supplementary file 1 — Additional file 1: Figure 1. Principal component analysis of 131 rice genotypes, with PC1, PC2 PC3 and PC4 classifying the whole germplasm into clusters. Figure 2. The pedigree groups A, B and C obtained from the rice breeding database according to their breeding history, the top one is the ancestor of the other below in the group. The available male sterile gene is indicated in braces with the name of each ancestral genotype. Figure 3. Top 1% selection sweeps on nine chromosomes indicating the genetic differentiation evaluated by Fst values among four populations, the red box in the Chromosome 2 indicated commonly identified and validated top selective sweep. Figure 4. The top enriched gene ontological function revealed Gene Ontology data bases for candidate genes from selective sweep analysis of conventional breeding lines versus pedigree group A (A), B (B) and C (C). [file 12870_2021_3062_MOESM1_ESM.doc]
