# Supplementary figures and images for: Genetic relationships and identification of core germplasm among rice photoperiod- and thermo-sensitive genic male sterile lines
Source: BMC Plant Biol. 2021 Jul 2;21:313. doi: 10.1186/s12870-021-03062-x (PMC8252326; doi:10.1186/s12870-021-03062-x)

# LD decay

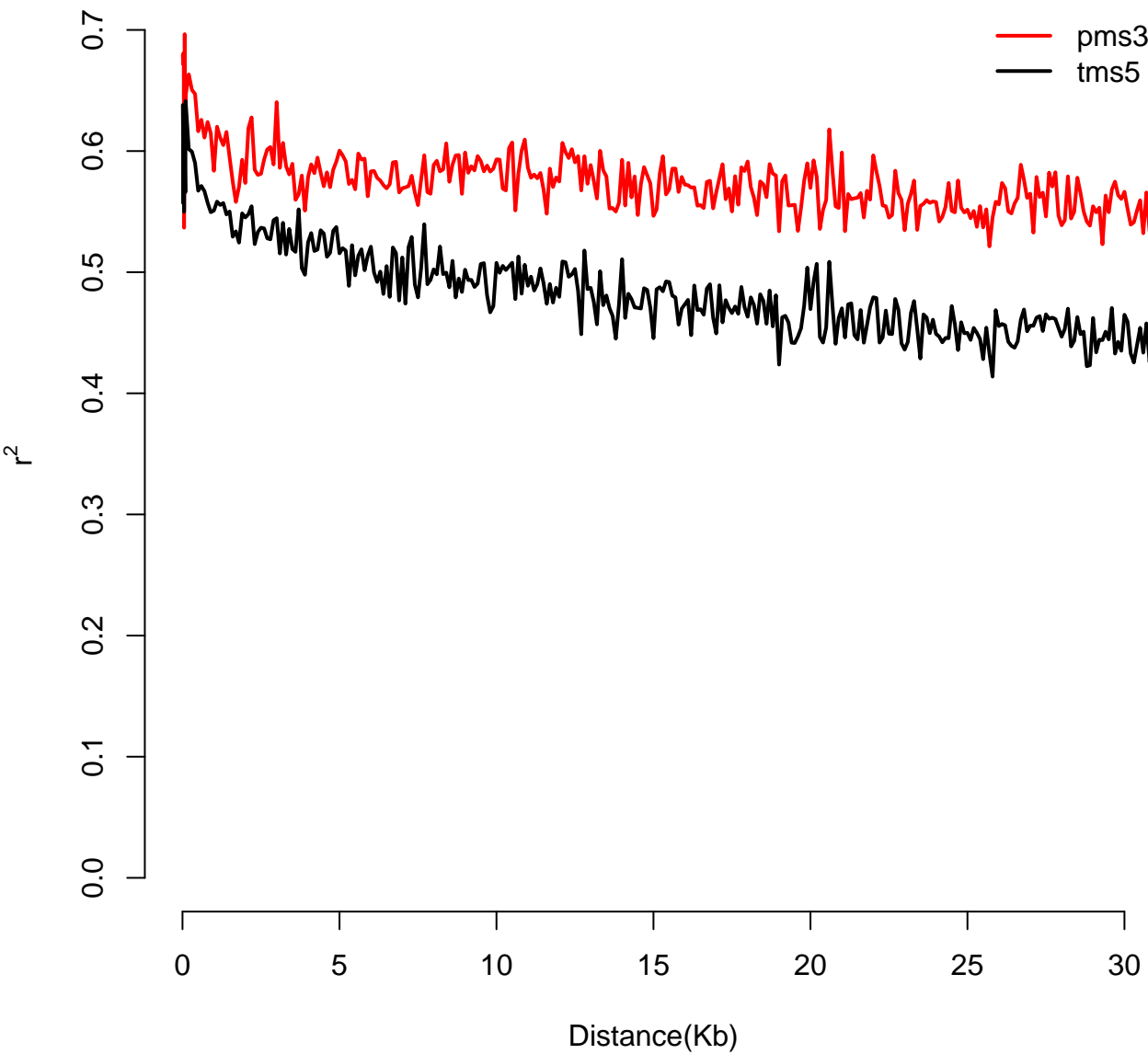

Supplement: Supplementary file 2 — Additional file 2: Figure 5. Linkage disequilibrium decay plot, the pairwise LD values (r2) plotted against the physical distance. [file 12870_2021_3062_MOESM2_ESM.pdf]
